# United States multicenter study of factors predicting the persistence of GH deficiency during the transition period between childhood and adulthood

**DOI:** 10.1186/1687-9856-2013-6

**Published:** 2013-02-13

**Authors:** Charmian A Quigley, Anthony J Zagar, Charlie Chunhua Liu, David M Brown, Carol Huseman, Lynne Levitsky, David R Repaske, Eva Tsalikian, John J Chipman

**Affiliations:** 1 Lilly Research Laboratories, Indianapolis, IN 46285, USA; 2 Allergan, Inc, Irvine, CA, 92612, USA; 3 Division of Pediatric Endocrinology, University of Minnesota, Minneapolis, MN, 55455, USA; 4 Children’s Mercy Hospital, Kansas City, MO, 64108, USA; 5 MassGeneral Hospital for Children, Boston, MA, 02114, USA; 6 Nationwide Children’s Hospital, Columbus, OH, 43205, USA; 7 University of Iowa, Children’s Hospital of Iowa, Iowa City, IA, 52242, USA; 8 Lilly Research Laboratories, Indianapolis, IN, 46285, USA

**Keywords:** Transition, Growth hormone deficiency, Hypopituitarism, Predictive value of tests, Child, Adolescent, Adult, Idiopathic

## Abstract

**Background:**

Many patients with childhood-onset growth hormone (GH) deficiency do not fulfill diagnostic criteria for GH deficiency (GHD) after attainment of adult height and may not require long-term GH treatment. Patients with history of idiopathic GHD (IGHD) pose the greatest management dilemma, as data regarding factors predictive of persistent GHD in this group are lacking.

**Objectives:**

The objective of this study was to assess potential predictors of persistent GHD in a US patient cohort during transition from childhood to adulthood, particularly in patients with history of IGHD.

**Methods:**

We studied 73 US patients with history of childhood-onset GHD screened at 21 US pediatric endocrine centers for a randomized clinical trial of GH replacement after attainment of adult height. The cohort comprised 42 boys/men and 31 girls/women aged14–22 years, who had received ≥1 year of GH treatment and had completed linear growth. The main outcome measures were sensitivity, specificity, positive and negative predictive values (PPV, NPV) of clinical and hormonal factors for persistent GHD (defined *a priori* in this study as peak GH < 5 μg/L).

**Results:**

For the cohort as a whole, the best predictors of persistent GHD (100% PPV) were history of organic hypothalamic-pituitary disorder or ≥2 additional pituitary hormone deficiencies (PHD). Best predictors of persistent GHD in patients with childhood history of IGHD were standard deviation scores (SDS) for serum insulin-like growth factor binding protein-3 (IGFBP-3) below -2.0, and for insulin-like growth factor-I (IGF-I) below -5.3 (measured ≥6 weeks after completion of GH treatment; PPV 100% for both), and age <4 years at original diagnosis (PPV 89%). IGF-I above -1.6 SDS had 100% NPV.

**Conclusions:**

US patients with an organic cause of childhood-onset GHD or ≥2 additional PHDs may not require GH stimulation testing to reconfirm GHD after completion of childhood treatment. In contrast, patients with idiopathic childhood-onset GHD almost invariably require retesting, as GHD persists in only a minority (those who were very young at initial diagnosis and those who have subnormal IGFBP-3 or extremely low IGF-I after completion of childhood treatment). Subnormal posttreatment IGF-I (<-2.0 SDS) lacked predictive power for persistent GHD, whereas IGF-I > -1.6 SDS was 100% predictive of GH sufficiency.

## Background

Growth hormone (GH) treatment of patients with GH deficiency (GHD) diagnosed in childhood has historically focused on maximizing adult height. However, this limited goal overlooks the importance of GH for completion and maintenance of somatic and metabolic maturation, including bone mineralization; accrual of lean body mass, with accompanying increases in muscle strength and exercise capacity; and changes in lipid metabolism [1-18]. Thus, there is now consensus that GH replacement is important for those patients with childhood-onset GHD who remain GH deficient after completion of linear growth [18-22].

Pharmacologic GH stimulation testing is generally recommended to confirm the diagnosis of persistent GHD during the childhood-to-adulthood transition, but this procedure requires interruption of GH therapy, is labor intensive, and is logistically challenging, given the scarcity of testing agents now available. In addition, provocative testing is invasive, has the potential for significant side effects, and produces inconsistent results that do not predict treatment response [19-24]. Because of these issues, several European studies have examined clinical and biochemical predictors of persistent GHD [25-30]. However, interpretation of the data is affected by factors such as the retrospective nature of most studies, interstudy differences in diagnostic criteria, and interassay variability. Furthermore, because previous studies have been performed in Europe, where diagnostic and treatment practices differ from US practices, the existing data may not be directly applicable to the largest group of children treated in the USA—those with idiopathic GHD (IGHD). Therefore, this study determined the prevalence of persistent GHD after attainment of adult height in a cohort of US childhood-onset GH-deficient patients during the transition period, with particular focus on those with IGHD, and examined the value of various factors as diagnostic predictors of persistent GHD.

## Methods

### Patients

This study screened 73 patients at 21 US institutions for entry to a randomized clinical trial of GH effects on bone and body composition in previously treated childhood-onset GH-deficient patients (efficacy and safety data have been reported [12,15]). The study was approved by the institutional review boards of participating institutions, and written informed consent was obtained from patients and/or their legal guardians.

Study entry criteria included: age 14–28 years; diagnosis of GHD during childhood/adolescence (either idiopathic or organic [i.e. due to a genetic or structural cause]); GH treatment ≥1 year, completed 6 weeks–5 years before screening; attainment of adult height (height velocity <1 cm/year); no history of spinal or total body irradiation, bone dysplasia, or significant systemic illness. Patients with additional pituitary hormone deficiencies (PHDs) were required to have received stable replacement therapy (thyroxine, glucocorticoids, sex steroids, vasopressin, as needed) for ≥6 months. The US cohort from this international study was selected for the analysis reported here because serum GH, insulin-like growth factor-I (IGF-I), and insulin-like growth factor binding protein-3 (IGFBP-3) concentrations for all US patients were measured at a central laboratory.

Baseline demographic data included etiology and age at diagnosis of childhood GHD, duration of previous GH treatment, presence of additional PHDs, age, and height and weight at retesting.

### Assessment of GH secretion

Screening for entry to the adult GH replacement trial included IGF-I and IGFBP-3 measurements followed by GH stimulation testing. A single stimulation test was sufficient for patients with history of multiple PHDs (MPHD); 2 tests were required for patients with history of isolated GHD. Protocol-preferred stimulation tests included insulin tolerance test (ITT), combined arginine/L-dopa test, and glucagon test. However, to represent the breadth of US pediatric endocrine practice, no specific testing protocol was mandated. Patients were eligible to enroll in the GH replacement trial if IGF-I was <1^st^ percentile for age/sex and peak GH was <5 μg/L. The GH threshold for definition of GHD was specified *a priori* in the protocol and is consistent with guidelines for diagnosis of GHD during the transition period [19-21]. Data from all US patients are included in this report, regardless of eligibility for the GH replacement trial.

### Laboratory analyses

IGF-I was measured by an IGFBP-blocked radioimmunoassay as described elsewhere *(*sensitivity 0.1 μg/L; intra- and interassay coefficients of variation [CV], 1.6% and 6.4%, respectively [31]). IGFBP-3 was measured by radioimmunoassay (sensitivity 0.13 mg/L; intra- and interassay CV, 1.9% and 9.2%, respectively [32]). Results were converted to standard deviation scores (SDS) using data for age/sex-matched controls from the same assays. GH was measured using an immunochemiluminometric assay specific for 22-kDa human GH [33]. All assays were performed centrally at Esoterix Endocrinology, Inc (Calabasas Hills, CA, USA).

### Statistical analyses

Statistical analyses were performed using the SAS software system (SAS Institute, Inc, Cary, NC). Because stimulated GH values were not normally distributed, the nonparametric Wilcoxon test was used to evaluate differences between GH-deficient *vs.* non–GH-deficient patients with respect to number of additional PHDs, serum IGF-I/IGFBP-3, age at original diagnosis, weight, and body mass index (BMI; kg/m^2^). The difference in peak GH among patients with 0, 1, ≥1, or ≥2 PHDs was examined using the nonparametric Kruskal-Wallis test. Relationships between peak GH and potential explanatory variables were assessed using Spearman correlation coefficients (r_s_). Summary data for continuous variables are presented as mean ± SD unless otherwise noted.

### Calculation of sensitivity, specificity, positive predictive value, and negative predictive value

Sensitivity, specificity, positive predictive value (PPV), and negative predictive value (NPV) were calculated to determine the utility of clinical and laboratory variables as screening tests for persistent GHD (defined as peak GH response <5 μg/L). Screening variables included etiology of childhood GHD (organic *vs.* idiopathic), age at childhood diagnosis, number of additional PHDs, and study entry values for weight, BMI, IGF-I, and IGFBP-3. Continuous variables (age, weight, BMI, IGF-I, and IGFBP-3 SDS) were tested to determine cut-off values predictive of GHD. Patients with values beyond the cut-off were classified as having a positive screening test (screen) for GHD. Patients with a positive screen who had maximum GH < 5 μg/L were designated as true positive (TP); patients with a positive screen who had peak GH ≥ 5 μg/L were designated false positive (FP); a negative screen accompanied by peak GH ≥ 5 μg/L was defined as true negative (TN); a negative screen with peak GH < 5 μg/L was defined as false negative (FN).

The following additional definitions were used: s*ensitivity* (of the screening test)*,* represent the probability of a positive screen among patients with GHD (i.e. proportion of GH-deficient patients correctly identified by the screen, calculated as TP/[TP + FN]); *specificity,* the converse of sensitivity, represents the probability of a negative screen among non–GH-deficient patients (proportion of non–GH-deficient patients correctly identified by the screen; TN/[TN + FP]); *PPV,* is the probability of GHD among patients with a positive test (proportion of patients with positive screen who were GH deficient; TP/[TP + FP]); *NPV, *is the probability of being non-GH deficient among patients with a negative screen (proportion of patients with negative screen who were non–GH deficient; TN/[TN + FN]). These calculations were determined for all patients (organic and idiopathic combined) and repeated separately for patients with IGHD.

## Results

### Historical and demographic data

**Figure 1:**
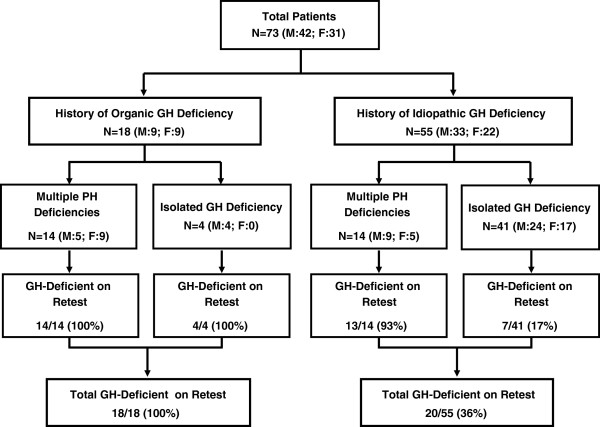
**Participant flow diagram. **Participant flow diagram of patients included in this study, showing numbers with history of organic *vs*. idiopathic GH deficiency, multiple pituitary hormone deficiencies *vs*. isolated GH deficiency, and numbers with confirmed GH deficiency on retesting. F = female; GH = growth hormone; M = male; N = number of patients; PH = pituitary hormone.

Of 73 patients (42 male, 31 female; ages 13.7–22.4 years), 18 had history of organic GHD, and 55 had history of IGHD (Figure 1, Table 1). Organic causes of GHD included craniopharyngioma (n = 6), glioma (n = 3), astrocytoma (n = 2), germinoma (n = 2), cranial irradiation (n = 2); 1 patient each had a history of medulloblastoma, septo-optic dysplasia, and pituitary hypoplasia.

**Table 1:** Demographic and diagnostic data

	**GHD at retest (peak GH <5 μg/L)**			Non-GHD at retest*	p values**	
**Variable**	**Organic n = 18 (M:9; F:9)**	**Idiopathic n = 20 (M:13; F:7)**	**Total n = 38 (M:22; F:16)**	**All patients n = 35 (M:20; F:15)**	**All: GHD vs. non-GHD**	**Idiopathic: GHD vs. non-GHD**
n (%) patients with isolated GH deficiency	4 (22)	7 (35)	11 (29)	62 (89)		
Age at diagnosis (yr)^1^	10.0 ± 2.2	4.5 ± 3.2	7.1 ± 3.9	10.5 ± 3.3	<0.001	<0.0001
	10.6 (6.3, 13.7)	4.6 (0.1, 11.6)	7.2 (0.1, 13.7)	11.0 (2.2, 16.9)		
Age at retest (yr)	17.9 ± 2.2	17.6 ± 1.8	17.7 ± 2.0	17.0 ± 1.6	0.08	0.17
	18.2 (14.1, 22.4)	17.6 (13.7, 21.9)	17.6 (13.7, 22.4)	16.7 (14.1, 20.2)		
Duration of childhood GH treatment (yr) ^1^	5.6 ± 2.9	11.4 ± 3.6	8.6 ± 4.4	5.5 ± 2.8	<0.01	<0.0001
	4.6 (2.0, 11.8)	12.2 (3.1, 16.7)	8.1 (2.0, 16.7)	4.7 (1.4, 12.7)		
Time off GH (yr)	1.7 ± 1.6	1.5 ± 1.0	1.6 ± 1.3	0.9 ± 0.6	0.01	<0.01
	1.2 (0.1, 5.0)	1.4 (0.2, 4.2)	1.3 (0.1, 5.0)	0.7 (0.2, 2.2)		
Weight (kg)	76.9 ± 18.8	75.5 ± 19.7	76.2 ± 19.0	60.5 ± 10.0	<0.001	<0.01
	74.6 (44.9, 119.3)	69.8 (47.1, 110.5)	74.2 (44.9, 119.3)	59.2 (42.5, 82.4)		
BMI (kg/m^2^)	27.5 ± 6.2	26.4 ± 5.5	26.9 ± 5.8	21.9 ± 2.9	<0.001	<0.01
	26.8 (15.1, 37.4)	26.2 (18.6, 37.4)	26.2 (15.1, 37.4)	21.0 (16.6, 29.8)		
BMI SDS	0.9 ± 1.6	0.9 ± 1.2	0.9 ± 1.4	0.1 ± 0.9	<0.001	<0.01
	1.4 (-4.4, 2.5)	1.4 (-1.8, 2.5)	1.4 (-4.4, 2.5)	0.2 (-2.3, 1.9)		
Number of additional PHDs	2.3 ± 1.7	1.5 ± 1.4	1.9 ± 1.6	0.0 ± 0.2	<0.0001	<0.0001
	2.5 (0.0, 4.0)	1.5 (0.0, 4.0)	2.0 (0.0, 4.0)	0.0 (0.0, 1.0)		
Peak GH (μg/L)	0.7 ± 0.9	0.5 ± 0.6	0.6 ± 0.8	15.2 ± 10.1	<0.0001	<0.0001
	0.2 (0.1, 3.0)	0.3 (0.0, 2.2)	0.2 (0.0, 3.0)	13.0 (5.0, 57.0)		
IGF-I (μg/L)	100 ± 67	123 ± 78	112 ± 73	309 ± 123	<0.0001	<0.0001
	86 (30, 265)	95 (20, 248)	93 (20, 265)	295 (117, 738)		
IGF-I SDS	-6.2 ± 2.4	-5.7 ± 2.7	-6.0 ± 2.5	-1.9 ± 1.4	<0.0001	<0.0001
	-6.0 (-9.8, –1.6)	-5.8 (-11.1, –1.6)	-5.8 (-11.1, –1.6)	-2.0 (-5.3, 1.5)		
IGFBP-3 (μg/L)	2.5 ± 1.0	2.8 ± 1.3	2.7 ± 1.2	3.8 ± 0.7	<0.0001	<0.01
	2.5 (1.1, 4.5)	2.4 (1.1, 5.9)	2.5 (1.1, 5.9)	3.8 (2.5, 5.4)		
IGFBP-3 SDS	-1.4 ± 1.6	-1.2 ± 1.9	-1.3 ± 1.7	0.4 ± 0.8	<0.0001	<0.01
	-1.3 (-4.5, 1.2)	-1.4 (-4.6, 2.4)	-1.3 (-4.6, 2.4)	0.4 (-1.2, 1.9)		

Values are means ± SD and median (minimum, maximum). *All patients who retested as non-GH deficient had idiopathic GH deficiency in childhood. **p values for comparisons of groups who were GH deficient vs. non-GH-deficient at retest were obtained from nonparametric Wilcoxon tests. ^1^Comparisons between organic vs. idiopathic patients with GHD at retest: p < 0.0001 for age at diagnosis and duration of childhood GH treatment; all others, nonsignificant.

Abbreviations: *BMI* = body mass index, *F *= female, *GH *= growth hormone, *GHD *= GH deficiency, *IGF-I *= insulin-like growth factor I, *IGFBP-3 *= insulin-like growth factor binding protein 3, *kg *= kilogram, *M *= male, *m*^*2 *^= meters squared, *n *= number, *PHDs *= pituitary hormone deficiencies, *SDS *= standard deviation score, *yr *= year.

Twenty-eight of 73 patients (38%) had ≥1 additional PHD (14/18 [78%] organic; 14/55 [25%] idiopathic). In order of prevalence these were: thyroid-stimulating hormone (TSH, n = 25 [34%]); gonadotropins (n = 20 [27%]); adrenocorticotropic hormone (n = 17 [23%]); vasopressin (n = 10 [14%]). Eight patients (11%) had 1 additional PHD, 5 (7%) had 2 additional PHDs, 6 (8%) had 3 additional PHDs, and 9 (12%) had 4 additional PHDs. The relationship between additional PHDs and likelihood of persistent GHD is reported below.

### GH stimulation retest results

The following GH stimulation tests were performed: arginine/L-dopa (48/73 [66%]); arginine alone (11/73 [15%]); ITT alone (7/73 [10%]); ITT/arginine (3/73 [4%]); 1 patient each was tested with ITT/clonidine, ITT/L-dopa, L-dopa alone, and an unspecified test. As shown in Figure 1 and Table 1, 38 of 73 patients had peak GH <5 μg/L at retest (male, 22/42 [52%]; female, 16/31 [52%]; organic, 18/18 [100%]; idiopathic, 20/55 [36%]); 37/38 (97%) patients with persistent GHD had severe GHD, with peak GH <2.5 μg/L. Of 20 patients with history of childhood IGHD confirmed as having persistent GHD (GH <5 μg/L), only 3 patients had peak GH values >1.0 μg/L at retest (1.5, 1.6, 2.2 μg/L). Patients with persistent GHD were younger at original diagnosis than those not reconfirmed as GH deficient, and at screening were significantly heavier and had lower posttreatment serum IGF-I and IGFBP-3 (Table 1).

### Predictors of persistent GH deficiency

#### All patients

The strongest predictor of persistent GHD was history of organic hypothalamic-pituitary disorder: 18/18 organic patients retested as GH deficient (100% PPV) *vs.* 20/55 (36%) of those with history of IGHD. However, sensitivity was low (47%) because history of IGHD did not preclude persistent GHD.

**Figure 2:**
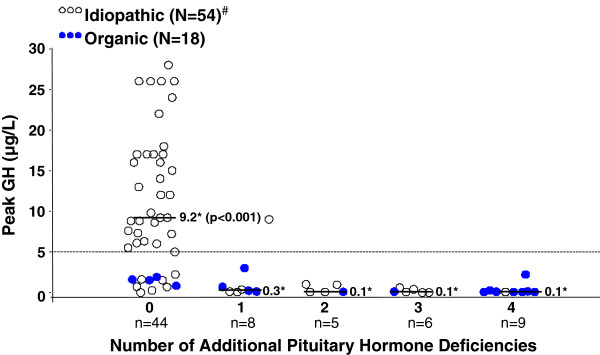
**Peak GH response according to number of additional pituitary hormone deficiencies (PHD). **Horizontal lines represent the median values of the peak stimulated GH concentrations for patients with 0, 1, 2, 3, and 4 additional PHDs; *p < 0.0001 for comparison of medians for the group with isolated GH deficiency (no additional PHDs) *vs. *all others. See “Results” for listing of stimulation tests used. ^#^To avoid compressing the vertical axis, 1 GH value of 57 μg/L (idiopathic patient) is not shown. GH = growth hormone; N = total number of patients in each category (organic *vs. *idiopathic); n = number of patients in each subgroup.

The second highly predictive finding was the presence of additional PHDs (Figure 2, Table 2). Of 28 patients with ≥1 additional PHD, 27 (96%) had GH < 5 μg/L at retest (13/14 [93%] idiopathic, 14/14 [100%] organic). Overall, GHD was reconfirmed in 24%, 88%, 96%, and 100% of patients with 0, 1, ≥1, and ≥2 additional PHDs, respectively (PPV 100% for ≥2 PHDs; Table 2). Peak stimulated GH (μg/L) was significantly lower in patients with ≥1 additional PHD than in those with isolated GHD (mean ± SD, median, range: 0.7 ± 1.8, 0.1, 0.0–9.0; *vs.* 11.9 ± 10.8, 9.2, 0.0–57.0; p < 0.001). However, presence of additional PHDs was not an essential feature of persistent GHD, as almost one-quarter of patients with history of isolated GHD had persistent GHD (overall, 11/45 [24%]; organic, 4/4 [100%]; idiopathic, 7/41 [17%]: Figure 1). As a corollary, 11/38 patients (29%) with reconfirmed GHD had childhood history of isolated GHD (organic and idiopathic combined).

**Table 2:** **Predictors of persistent GH deficiency: *all patients *(n = 73)**

	**History of organic disease**	**≥2 extra PHDs**	**≥1 extra PHD**	**IGF-I < -5.3 SDS**	**IGF-I < -3.0 SDS**	**IGF-I < -2.0 SDS**	**≥1 extra PHD and IGF-I < -2.0 SDS**	**IGFBP-3 < -2.0 SDS**
**Positive Predictive Value (%)**	100	100	96	100	84	65	100	100
**Negative Predictive Value (%)**	64	66	76	71	83	84	75	59
**Specificity (%)**	100	100	97	100	83	46	100	100
**Sensitivity (%)**	47	53	71	63	84	92	68	37

Abbreviations: *IGF-I* = insulin-like growth factor I, *IGFBP-3* = insulin-like growth factor binding protein 3, *n* = number, *PHD* = pituitary hormone deficiency, *SDS* = standard deviation score.

Because history of organic hypothalamic-pituitary disorder had 100% PPV for persistent GHD during transition, results for predictive value of IGF-I SDS, IGFBP-3 SDS, age at original diagnosis, body weight, and BMI are presented below only for the 55 patients with history of IGHD.

#### Patients with idiopathic GH deficiency

##### IGF-I and IGFBP-3

**Figure 3:**
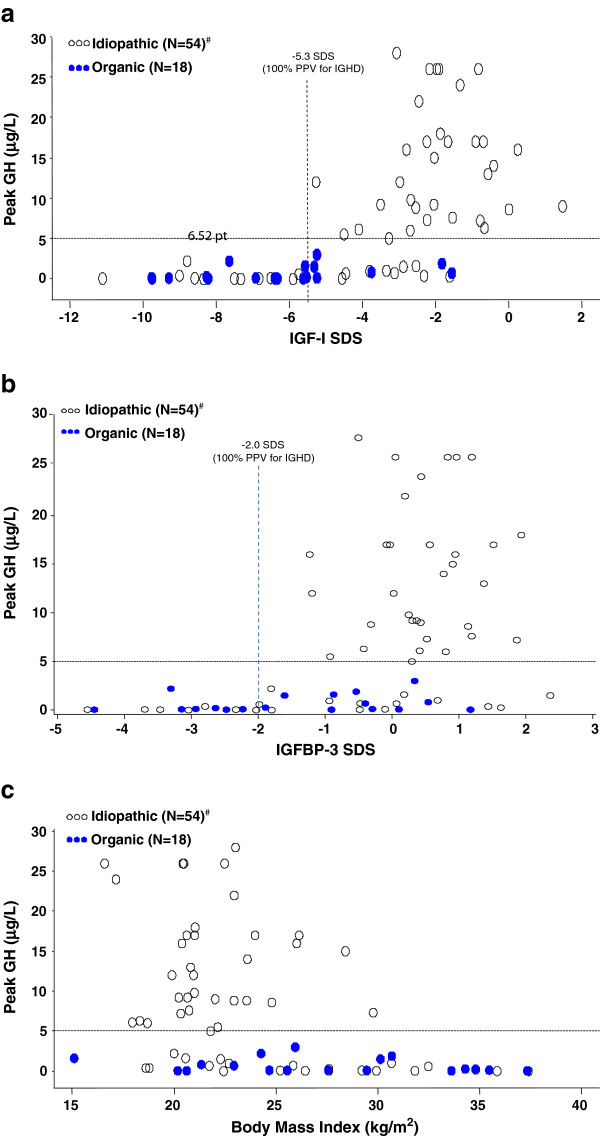
**a. Relationship between IGF-I SDS and peak GH response. **Dashed vertical line represents the IGF-I threshold of -5.3 SDS, which provides 100% PPV for the diagnosis of persistent GH deficiency in patients with IGHD. IGF-I = insulin-like growth factor –I; **b. Relationship between IGFBP-3 SDS and peak GH response. **Dashed vertical line represents the IGFBP-3 threshold of -2.0 SDS, which provides 100% PPV for the diagnosis of persistent GH deficiency in patients with IGHD. IGFBP-3 = insulin-like growth factor binding protein 3; **c. Relationship between body mass index and peak GH response. **For the idiopathic group, Spearman r = -0.39, p = 0.003. There was no significant correlation for the organic group. *Notes and abbreviations: *See “Results” for listing of stimulation tests used; ^#^One outlier idiopathic patient with a peak GH of 57 μg/L was excluded to avoid undue influence on the correlation and compressing the vertical axis. GH = growth hormone; IGHD = idiopathic GH deficiency; N = total number of patients in each category (organic *vs. *idiopathic); PPV = positive predictive value; SDS = standard deviation score.

As reported above, 20 of 55 (36%) patients with history of IGHD had peak <5 μg/L at retest. Mean serum IGF-I concentrations were subnormal (both as absolute values and as SDS) in patients with peak GH < 5 μg/L (Table 1); however, the range of IGF-I SDS values was wide (-1.6 to -11.1; Table 1, Figure 3a). Mean IGF-I SDS was lower for female than for male patients with persistent GHD (-7.68 ± 2.54 vs. -4.65 ± 2.19, p = 0.04). There was a strong correlation between IGF-I SDS and peak GH (n = 54, r_s_ = 0.67, p < 0.0001) primarily driven by the lower IGF-I SDS values. For idiopathic patients with peak GH < 5 μg/L at retest, the correlation was modest but did not quite reach statistical significance: n = 20, r_s_ = 0.43, p = 0.06; for those with GH ≥ 5 μg/L the correlation was lower and non-significant: n = 34, r_s_ = 0.20, p = 0.26.

Although two thirds of patients with history of IGHD (36/54 [67%]; value missing for 1 patient) had subnormal serum IGF-I (<-2.0 SDS) at retesting, this threshold did not discriminate well between those who retested with peak GH above (n = 35) or below (n = 20) 5 μg/L (specificity 50%; Table 3). Therefore, different threshold values of IGF-I SDS were examined to determine the cut-off that provided optimal predictive power. Whereas only 19/36 (53%) of idiopathic patients with IGF-I < -2.0 SDS had peak GH < 5 μg/L at retest, PPV increased to 73% at -3.0 SDS, 81% at -4.0 SDS, and 100% at -5.3 SDS (Table 3). However, at this very low cutoff, sensitivity was only 55% because 9 idiopathic patients who retested as GH deficient had IGF-I SDS greater than this threshold (Figure 3a). Notably, only 1 idiopathic patient who retested as GH deficient had IGF-I > -2.0 SDS, and none had IGF-I > -1.6 SDS; thus IGF-I > -1.6 SDS had 100% NPV for GHD (Table 3, Figure 3a).

**Table 3:** **Predictors of persistent GH deficiency in patients with history of *idiopathic GH deficiency *(n = 55)**

	**≥1 cextra PHD**	**≥2 extra PHD**	**IGF-I < -5.3 SDS**	**IGF-I < -4.0 SDS**	**IGF-I < -3.0 SDS**	**IGF-I < -2.0 SDS**	**IGF-I > -1.6 SDS**	**IGFBP-3 < -2.0 SDS**	**Age <4 yr at original Dx**	**≥1 PHD + IGF-I < -2.0 SDS**	**IGF-I < -2.0 SDS + age <4 yr at original Dx**
**Positive Predictive Value (%)**	93	100	100	81	73	53	49	100	89	100	100
**Negative Predictive Value (%)**	83	78	79	82	88	94	100	73	76	83	75
**Specificity (%)**	97	100	100	91	82	50	38	100	97	100	100
**Sensitivity (%)**	65	50	55	65	80	95	100	35	42	65	40

Abbreviations: *Dx* = diagnosis, *GH* = growth hormone, *IGF-I* = insulin-like growth factor I, *n* = number, *PHD* = pituitary hormone deficiency, *SDS* = standard deviation score, *yr* = year.

In general, mean IGFBP-3 concentrations were closer to average for age/sex than IGF-I in the idiopathic cohort (Table 1), but were somewhat lower for female than male patients (for patients with persistent GHD: female, -1.86 ± 2.39; male, -0.77 ± 1.56; p = 0.28). Subnormal IGFBP-3 was more predictive of persistent GHD than subnormal IGF-I in this group, as all idiopathic patients with IGFBP-3 < -2.0 SDS had peak GH < 5 μg/L on retest (PPV 100%; Table 3, Figure 3b).

##### Age at original diagnosis

**Figure 4:**
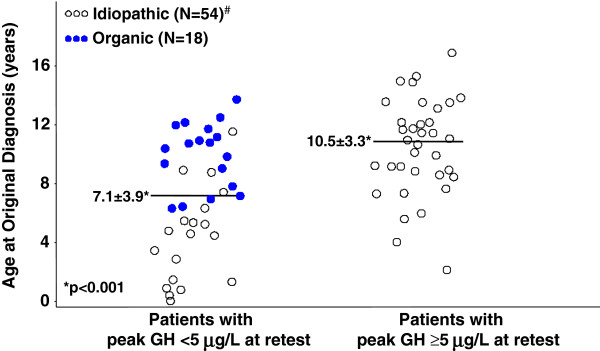
**Age at original childhood diagnosis of GH deficiency. **Distribution of age at original diagnosis for patients who retested as GH deficient (left) *vs. *those who retested as GH sufficient (right). Within the persistently GH-deficient group, patients with history of idiopathic GH deficiency were significantly younger at diagnosis than those with history of organic GH deficiency (Table 1). Horizontal lines represent mean ages at initial diagnosis for patients with history of IGHD. GH = growth hormone; N = total number of patients in each category (organic *vs.* idiopathic).

Young age (<4 years) at diagnosis of childhood IGHD was a strong predictor of persistent GHD in this group, with 97% specificity and 89% PPV (Table 3). On average patients with history of IGHD who later retested as GH deficient were less than half the age at original diagnosis of those who retested as non–GH deficient (4.5 ± 3.2 *vs*. 10.5 ± 3.3 years, p < 0.0001; Table 1, Figure 4). IGHD patients with persistent GHD therefore had received GH treatment for twice as long as those who retested as non–GH deficient and those with organic GHD (Table 1).

##### Sex, body weight, and BMI

The proportion of patients with history of IGHD who had persistent GHD at retest was similar for male and female patients (13/33 [39%] *vs.* 7/22 [32%]). Body weight and BMI at retest were significantly greater in patients with persistent GHD than in those with peak GH ≥ 5 μg/L (p < 0.01), demonstrating modest but significant inverse correlations with peak GH (BMI *vs.* peak GH: n = 54, r_s_ = -0.39, p = 0.003) and with IGF-I SDS (BMI *vs.* IGF-I SDS: *n* = 54, r_s_ = -0.31, p = 0.02). Although there was a wide range of BMI values (18.6–37.4 kg/m^2^) in idiopathic patients with persistent GHD, all patients with BMI >30 kg/m^2^ had peak GH <5 μg/L at retest (Figure 3c).

Because overweight and obesity may blunt GH secretion even in non–GH-deficient individuals, we specifically evaluated all idiopathic patients who had GH stimulation test results consistent with persistent GHD and had BMI >25 kg/m^2^ (World Health Organization definition of overweight) at retesting. Of 12 such patients, 10 had additional PHDs, and therefore had independent factors strongly predictive of persistent GHD (93% PPV), irrespective of BMI. The 2 overweight patients with isolated IGHD whose GH responses could potentially have been blunted by being overweight, were only mildly overweight and had peak GH values <1μg/L (patient 1: BMI 26 kg/m^2^, peak GH 0.70 μg/L; patient 2: BMI 28 kg/m^2^, peak GH 0.26 μg/L). Therefore, given these extremely low peak GH concentrations, it seems unlikely that either of these patients was misclassified as GH deficient due to obesity-related blunting of GH secretion. Overall, neither weight nor BMI was a good predictor of persistent GHD (e.g. PPV 36% for BMI 38 kg/m^2^).

## Discussion

Since the early 1990s the role of GH in many physiologic processes in adulthood has become clearer, and the importance of GH replacement for GH-deficient adults is well established [19-21,34]. Many studies have demonstrated deficits in somatic and metabolic maturation in GH-deficient individuals untreated during the transition period [1-4,6-17]. However, the determination of precisely which patients require ongoing GH therapy has been less clear, as many patients treated for childhood GHD do not fulfill diagnostic criteria for adult GHD after completion of linear growth. This finding may reflect a number of factors, including differences in diagnostic criteria for GHD in childhood *vs*. adulthood, lack of reproducibility of GH stimulation tests, and perhaps sex steroid–mediated maturational changes in hypothalamic control of GH secretion during puberty [23,35-37]. Consequently, retesting GH secretion in adolescents and young adults with childhood-onset GHD is generally recommended [19-22]. However, such testing requires interruption of GH therapy, and the results vary by protocol, secretagogue, and GH assay; lack reproducibility; and do not predict treatment response [23]. Furthermore, the increasingly limited availability of many agents for which GH stimulation testing protocols are established (e.g. arginine, GH-releasing hormone, L-dopa) leaves few options other than ITT, which requires physician presence because of the risk of complications such as seizures as a result of significant hypoglycemia [24,33]. Therefore, this study aimed to provide a rational basis for GH stimulation retesting in US patients by examining factors predictive of persistent GHD in a cohort of 73 patients with history of childhood-onset GHD who underwent centralized measurements of IGF-I, IGFBP-3, and GH after completion of childhood treatment. Because of limited published information, particular attention was focused on factors predictive of persistence in patients with history of IGHD, the most common form of childhood GHD treated in the USA.

Our finding that 100% of US patients with history of organic GHD had persistent GHD confirms previous European reports [26,27,30,38,39]. Similarly, we found a very high prevalence of persistent GHD in patients with ≥1 additional PHD (96% PPV) [25,29,40-42]. Thus it appears that despite potential differences between US and European physicians with regard to diagnosis and treatment of childhood GHD, the key factors associated with its persistence appear consistent across these geographies. The single patient with an additional PHD (TSH) who did not fulfill the study definition of GHD may nevertheless have a partial GH secretory defect because peak GH response to arginine/L-dopa was 9.0 μg/L. Other studies have concluded that such patients may have a milder form of GH “insufficiency” [29,43-45]. As GH is usually the first anterior pituitary hormone affected by pathological insults, there is a biological rationale to suspect that patients with ≥1 additional PHD will likely have persistent GHD [46,47]*.*

Organic etiology of GHD and presence of additional PHDs reflect the severity of hypothalamic-pituitary dysfunction, so it is not surprising that severe GHD persisted in almost all such patients; provocative GH retesting thus appears unnecessary in patients with organic disease [29,38,39,42]. Instead, GH potentially could be continued uninterrupted through the transition period (with appropriate dosage adjustment) to avoid the adverse changes in body composition, lipid profile, and cardiac function that may develop following discontinuation of GH [1-4,6-17]*.* Furthermore, patient care could potentially be improved by providing the family with a clear expectation at the initiation of childhood treatment, of the likelihood that GH treatment will be required in adulthood. 

Although only half of our patients with MPHD had a childhood diagnosis of organic disease, some patients whose MPHD was labeled “idiopathic” may, in fact, have had an undiagnosed genetic disorder. This is suggested in other studies by the presence of mutations in genes encoding pituitary transcription factors, most commonly *PROP1,* in up to half of patients with an original diagnosis of idiopathic MPHD [37,48-51]*.* Furthermore, up to one-quarter of children with isolated GHD may have detectable genetic defects [49,52,53]. Thus, genetic studies should be obtained whenever possible in any patient with MPHD or early-onset isolated GHD, because presence of a mutation would obviate the need for GH stimulation retesting after childhood treatment, and allow such patients to continue replacement therapy uninterrupted. Similarly, although our study did not include magnetic resonance imaging (MRI) assessment, MRI anomalies have been reported as a significant predictor of persistent GHD during transition [27,37,41,54], and certain MRI findings may indicate a genetic basis for hypothalamic-pituitary disorders [55-57]*.*

In contrast to those with organic hypothalamic-pituitary dysfunction, patients with childhood IGHD present a substantial diagnostic dilemma, and prior studies have not evaluated predictive factors for persistent GHD in this specific population. Moreover, as idiopathic patients represent the majority of recipients of childhood GH treatment in the USA [58-60]*,* they constitute the bulk of the clinical load for US pediatric endocrinologists. Therefore, our study specifically examined factors predictive of persistent GHD in this subgroup. Only about one-third of idiopathic patients (36%) retested as GH deficient; this was true for even fewer patients with isolated IGHD (17%). The low rate of persistent GHD in our US idiopathic cohort is similar to the rates reported in Belgian, British, and French studies, in which 15%–24% patients with childhood isolated IGHD remained GH deficient when retested [26,40,61]. However, our results differ notably from those of an Italian study in which 52%–65% of young adults with isolated IGHD were GH deficient on retest*,* likely reflecting the fact that about one-third of patients in the Italian study had severe childhood GHD [39].

Apart from the presence of additional PHDs, the strongest independent predictor of persistent GHD in our idiopathic cohort was the finding of IGFBP-3 below -2.0 SDS, which had 100% PPV for persistent GHD. In contrast, a subnormal IGF-I value (i.e. <-2.0 SDS) was not prognostically helpful in those with history of IGHD, as only half of such patients retested as GH deficient. However, an extremely low IGF-I (<-5.3 SDS) provided 100% PPV; in addition, the combination of IGF-I SDS below -2.0 and young age at original diagnosis of IGHD was strongly predictive of persistent GHD. Our finding of lack of predictive power of subnormal IGF-I contrasts with the good concordance between IGF-I and peak GH reported in European studies [25-27,55], perhaps reflecting the typically greater severity of GHD in European children, differences in agents and diagnostic cut-points used for GH testing, and time between discontinuation of GH and retesting (as GHD may manifest after increasing time off treatment [43,44]). Furthermore, IGF-I secretion is controlled by other factors in addition to GH, such as nutritional status and sex steroid milieu [32,62,63]. Perhaps more importantly, IGF-I may provide a good screen for GH *sufficiency,* as 100% of idiopathic patients who had IGF-I > -1.6 SDS were GH sufficient on retest (100% NPV for GHD). Patients with IGF-I SDS values above this level after discontinuation of GH treatment could be spared the invasive process of GH stimulation retesting after completion of childhood therapy, as all would be expected to be GH sufficient, and instead could be followed clinically.

The other useful predictor of persistent GHD in the idiopathic cohort was age <4 years at original diagnosis (specificity 97%, PPV 89%), likely reflecting the fact that growth failure occurs earlier in children with more severe GHD [29]. Consequently, families of children who are very young at initial diagnosis of IGHD should be forewarned of the likelihood of its permanence.

This study has a number of potential limitations. First, no direct comparison of GH stimulation test results at the time of childhood diagnosis versus results on retest in the present study could be made because initial testing was performed at the individual institutions and not at a central laboratory. For the same reason, we were unable to assess the predictive value of a number of other clinically relevant parameters, such as pretreatment IGF-I, height SDS, height velocity, or height gain in response to childhood treatment. Second, the single cut-point of 5 μg/L defined in the protocol to represent the threshold for GH deficiency irrespective of the testing agent used, may be considered to lack precision; a subsequent study in patients with *adult-onset* GHD (conducted after our study was designed and implemented) indicates that different diagnostic thresholds are appropriate for different agents [33]. However, evidence for the appropriateness of this approach is lacking for patients in the transition period, as noted by consensus statements from endocrine societies [19-21]. Third, because our study population comprised patients screened for aGH replacement trial, the cohort may represent the more severe end of the US childhood GHD spectrum, and persistent GHD may be less likely in milder cohorts. Nevertheless, our finding that only 17% of patients with history of isolated IGHD had persistent GHD is consistent with European data for this subgroup. Fourth, IGF-I assays have substantial interlaboratory variability, so the very low IGF-I SDS values predictive of persistent GHD in our study may not be applicable to IGF-I measured elsewhere. Fifth, obesity is associated with blunted GH response to stimulation, even in non–GH-deficient individuals [64], leading to potential bias toward overdiagnosis of GHD. Thus the peak GH threshold of 5 μg/L used for diagnosis of GHD in this study may be inadequately stringent for obese patients (BMI > 30 kg/m^2^) [20]. Nevertheless, as all obese patients in this study had additional PHDs, misdiagnosis due to obesity-related blunting of GH secretion seems unlikely. Finally, it is acknowledged that no single study can provide comprehensive guidelines for the broad range of patients treated and followed in different clinical settings, and assessment should be individualized for each patient.

## Conclusions

This US study demonstrates that patients with an organic basis for childhood-onset GHD and those with ≥2 additional PHDs may not require GH stimulation testing after completion of linear growth for confirmation of persistent GHD and potentially could continue GH treatment without interruption. However, as most children treated in the USA have an idiopathic, isolated form of GHD, the majority will likely not require GH treatment during adulthood. In patients with history of IGHD, the strongest predictor of persistent GHD was subnormal IGFBP-3 SDS (<-2.0 SDS), whereas subnormal IGF-I (<-2.0 SDS) lacked predictive power. Conversely, posttreatment IGF-I > -1.6 SDS was predictive of GH sufficiency. Therefore, unless IGF-I is extremely low (<-5.3 SDS) accompanied by subnormal IGFBP-3 (<-2.0 SDS), patients with IGHD should undergo GH retesting after completion of childhood treatment.

## Competing interests

This study was sponsored by Eli Lilly and Company (Indianapolis, IN). In compliance with the Uniform Requirements for Manuscripts, established by the International Committee of Medical Journal Editors, the sponsor did not impose any impediment, directly or indirectly, on the publication of the results of this study.

## Authors’ contributions

CAQ and JJC conceived the objectives questions and analyses reported in this manuscript; CAQ coordinated the study and manuscript development, and drafted the manuscript; AJZ and CCL participated in the design of the analyses and performed the statistical analyses; DMB, CH, LL, DRR, and ET revised the manuscript for intellectual content. All authors read and approved the final manuscript.
